# Dispersal and Gene Flow Among Potential Spawners: Source–Sink Structure Among Populations of Anadromous Brown Trout Exposed to Multifaceted Anthropogenic Impacts

**DOI:** 10.1111/eva.70130

**Published:** 2025-07-08

**Authors:** K. L. Hawley, J. Thaulow, H. A. Urke, T. Kristensen, N. J. Barson, T. O. Haugen

**Affiliations:** ^1^ Norwegian Institute for Water Research Oslo Norway; ^2^ Faculty of Environmental Sciences and Natural Resource Management The Norwegian University of Life Sciences Ås Norway; ^3^ AquaLife R&D Trondheim Norway; ^4^ Faculty of Biosciences and Aquaculture Nord University Bodø Norway; ^5^ Faculty of Biosciences Norwegian University of Life Sciences Ås Norway

**Keywords:** acoustic telemetry, demography, genetic assignment, homing, Leslie matrix, metapopulation, migration, philopatry, *Salmo trutta*, straying

## Abstract

Dispersal impacts individual fitness and influences local dynamics, stability and adaptation in interconnected populations. Anadromous salmonid fishes are renowned for their precise homing and adaptations to local aquatic environments, while navigating between multiple connected habitats. However, recent studies have demonstrated considerable straying among systems, generating metapopulation dynamics among connected subpopulations or demes. Salmonids constitute valuable economic and ecological resources, yet many populations are declining due to multifaceted anthropogenic‐induced disturbances. This context of reduced populations inhabiting altered environments may impact both population viability and dispersal. To explore if metapopulation processes are present among impacted neighbouring populations of anadromous brown trout ( *Salmo trutta* ), a 4‐year study of individual (*N* = 84) dispersal behaviour (using biotelemetry) and genetic analysis was conducted in four populations, connected by an extensive (> 200 km), semi‐enclosed fjord system, Sognefjorden, Norway. To estimate the demographic status of each study population, life‐table matrices were built, from which a potential source–sink structure among demes could be identified. Sognefjorden brown trout formed a metapopulation consisting of multiple sink populations, primarily supplemented from a single source. Only one population exhibited intrinsic growth (i.e., *λ* > 1), with excess recruits in this population attributed to high survival within the fjord. Among potential spawners, dispersal movements were performed by 55% of the total population, with individual age and migration extent affecting the probability of this behaviour. Successful dispersal (straying) was performed by 25% of the total spawning population. The extensive hydroscape generated directional gene flow from the innermost to outermost populations, with the highest rates observed among neighbouring populations. Although most dispersal resulted in unsuccessful spawning events and/or was not intended for spawning (e.g., conducted for overwintering purposes), connectivity among population demes was significant. This connectivity likely enhances the overall resilience of the metapopulation to variation and shifts in contemporary conditions within the fjord.

## 1 Introduction

Dispersal, the movement of individuals between populations, is a common ecological process, underpinning gene flow. Dispersal has consequences for individual fitness but may also influence the local dynamics, stability and adaptation of a set of populations (Haugen et al. 2006). Due to this link between dispersal, gene flow and population dynamics, understanding its causes and consequences is necessary to predict and manage population responses to environmental change (Bowler and Benton 2005; Morales et al. 2010; Cayuela et al. 2018).

Dispersal can be selected for if it results in reduced competition, kin interactions, inbreeding and buffers against habitat stochasticity (Bowler and Benton 2005; Crespo‐Miguel et al. 2022). From a behavioural perspective, dispersal is distinct from migration, which involves predictable spatial and temporal movements of individuals among breeding, foraging or refuge habitats (Dingle and Drake 2007; Lucas and Baras 2008). However, dispersal and migration are connected, with dispersal thought to be more prevalent among migratory species and life‐history types (Östergren and Nilsson 2012). Anadromous salmonids are known for their remarkable philopatric or homing abilities, often undertaking extensive migrations from shared marine feeding habitats to freshwater breeding habitats of their birth. This behaviour leads to genetic differentiation into distinct freshwater populations based on spawning sites and juvenile habitat in tributaries, rivers and lakes (Hasler et al. 1978; Ferguson et al. 2019). Natal philopatry acts as an evolutionary mechanism that enhances individual fitness by increasing the likelihood of finding suitable partners and habitats during reproduction, as well as fostering the development of local adaptations (Keefer and Caudill 2014; Mobley et al. 2019). Consequently, dispersal is often overlooked in salmonids, with dispersal or ‘straying’ to river systems not of their birth, conventionally considered a maladaptive exceptional event, due to some failure of imprinting or homing (Schtickzelle and Quinn 2007; Birnie‐Gauvin et al. 2019).

Landscape features may promote or limit dispersal (Baguette et al. 2013), with dispersal more frequently observed between nearby populations (Wang and Bradburd 2014). In hierarchical, dendritic habitats such as river networks or waterways, connectivity depends upon the position of a given habitat along the river network (Fagan 2002; Tonkin et al. 2018). This dependence can shape patterns of intraspecific genetic variability across space (e.g., isolation by distance; Wright 1943; Slatkin 1993). Connectivity among populations is often considered under the metapopulation paradigm, which defines a subdivided group of discrete populations (demes) that persist in a balance between migration, extinction and recolonisation (Hanski 1998). When connected habitats differ in quality, this can generate variable rates of population growth and stability among demes, creating a source–sink structure within a metapopulation.

Under the metapopulation paradigm, source habitats exhibit intrinsic positive growth (reproduction > mortality) and sinks negative growth (reproduction < mortality). Thus, sink populations rely on immigration from source populations for persistence (Pulliam 1988; Kawecki 2004; Schtickzelle and Quinn 2007). If the source population's compensatory reserve remains diminished over time, or if the sink population's habitats cannot sustain the population despite juvenile recruitment, the sink population will be lost (Kaeding 2023). Thus, if ongoing alterations occur over sustained periods (e.g., selective harvesting, climate change, migration barriers and habitat degradation) act towards either of these scenarios, sink populations would be eliminated according to their susceptibility, until only the source population remained (Kaeding 2023). Consequently, dispersal and metapopulation dynamics can weaken the link between local abundance and local demography (Hindar et al. 2004; Kawecki 2004; Cayuela et al. 2018). For example, a habitat with poor local reproduction but high abundance due to immigration from nearby populations would be misinterpreted as high‐quality if the importance of dispersal was overlooked (Schtickzelle and Quinn 2007). Dispersal is therefore important to consider when predicting population responses to anthropogenic disturbances at both large scales (e.g., climate‐induced alterations) and local scales (e.g., habitat fragmentation), with anadromous salmonids significantly impacted due to their dependence on multiple habitats and connectivity among them (Webster et al. 2002).

Due to its extreme ecological adaptability, the salmonid fish brown trout ( *Salmo trutta* ) is widely distributed throughout freshwater systems (Jonsson and Jonsson 2017; Nevoux et al. 2019). However, there are concerns about its conservation status under a range of anthropogenic stressors (ICES 2013). The main disturbances affecting brown trout include overfishing, habitat degradation (e.g., hydropower development, riverbed regulations, in‐river barriers), aquaculture, water pollution and climate change (Ayllón et al. 2016; Thorstad et al. 2016; Fiske et al. 2024), as well as genetic introgression from widespread stocking with non‐native strains (Hansen 2002; Hansen et al. 2009). In its anadromous form (i.e., sea trout), the iteroparous (i.e., spawning more than once over a lifetime) brown trout may undertake repeated migrations between freshwater, brackish and marine waters. This requires adaptation to local conditions while retaining the capability to cope with highly variable environments (Østergaard et al. 2003; Nevoux et al. 2019; Bekkevold et al. 2020). Recent research has observed that dispersal among anadromous brown trout populations may be relatively prevalent (Ayllón et al. 2006; Östergren et al. 2012; Masson et al. 2018; Mikheev et al. 2021; Källo, Baktoft, Kristensen, et al. 2022), with evidence suggesting that decision‐based processes aimed at maximising individual fitness can affect dispersal rates (Finlay et al. 2020; Källo, Baktoft, Birnie‐Gauvin, and Aarestrup 2022). For example, habitat quality, connectivity and size (Østergaard et al. 2003; King et al. 2020; Chat et al. 2022; Källo et al. 2024), as well as individual life‐history characteristics (e.g., duration of sea‐sojourn, Källo, Baktoft, Kristensen, et al. 2022; Källo et al. 2023) have been linked to dispersal probability, with dispersal behaviour shown to change over the lifetime of an individual (Källo, Baktoft, Birnie‐Gauvin, et al. 2022).

Dispersal patterns play an important role in the distribution of genetic variation within and among populations (Cayuela et al. 2018). Consequently, genetic information can be used to describe connectivity between populations and the impacts of individual dispersal events upon genetic diversity (Paetkau et al. 2004; Lemopoulos et al. 2019). When integrated with biotelemetry, a method which provides spatial–temporal information of marked individuals in situ (e.g., Lennox et al. 2017), genetic data can be used to quantify the population‐level consequences of the observed individual movements (Cayuela et al. 2018; Müller et al. 2023). In this study, we combined genetic and biotelemetry data of individual anadromous brown trout from four adjacent populations that share access to the same marine feeding habitat. By integrating these approaches, we aimed to describe the origin and destination of dispersal movements among potential spawners, rather than solely presenting rates of dispersal. We also aimed to quantify the relative reproductive success of immigrants and residents, and the resulting consequences in terms of gene flow and population connectivity within a spatially structured fjord system. When combined with age‐structured models of projected population demography and persistence (Caswell 2000), dispersal rates can be used to identify potential source–sink states under the metapopulation paradigm (Pulliam 1988; Furrer and Pasinelli 2016).

The sampled rivers flow into Sognefjorden, the world's longest fjord system supporting anadromous brown trout populations, located on the west coast of Norway. The fjord is semi‐enclosed and extends more than 200 km from the fjord mouth to the innermost river, resulting in virtual isolation from neighbouring fjord populations of brown trout (Skaala 1992). Sognefjorden was famed for its high productivity and substantial catches of large anadromous fishes (brown trout and Atlantic salmon, *Salar salar*). However, these populations have significantly declined due to multi‐faceted anthropogenic impacts which have simultaneously adversely altered the freshwater and fjord environments. Overfishing, habitat alterations and migration barriers (due to hydropower regulations) as well as high densities of salmon lice ( *Lepeophtheirus salmonis* ), sourced from open net pen salmon farms within the fjord, have all contributed to the decline of Sognefjorden's brown trout (Fiske et al. 2024). This context of small populations inhabiting altered environments may impact both the population viability and dispersal behaviour of brown trout within Sognefjorden (Østergaard et al. 2003; Bekkevold et al. 2020). Despite the extensive distances between study populations, we anticipated that dispersal would be relatively prevalent among anadromous brown trout in Sognefjorden, with life‐history models indicating limited intrinsic population growth.

In this study, we quantify the degree of dispersal movement among potential spawners of anadromous brown trout. We test the hypothesis that individual traits (length, age, sex and extent (distance) of sea‐sojourn) affect the probability of undertaking dispersal movements (H_1_). Secondly, we quantify the degree of effective gene flow among populations and test the hypothesis that the landscape or *hydroscape* (distance among rivers, location of river within the fjord system) affects the probability of evolutionary dispersal (or straying) among study populations (H_2_). Finally, we generate estimates of total evolutionary dispersal to identify if a source–sink metapopulation structure operates among populations of anadromous brown trout in Sognefjorden (H_3_).

## 2 Materials and Methods

### 2.1 Study System

**FIGURE 1:**
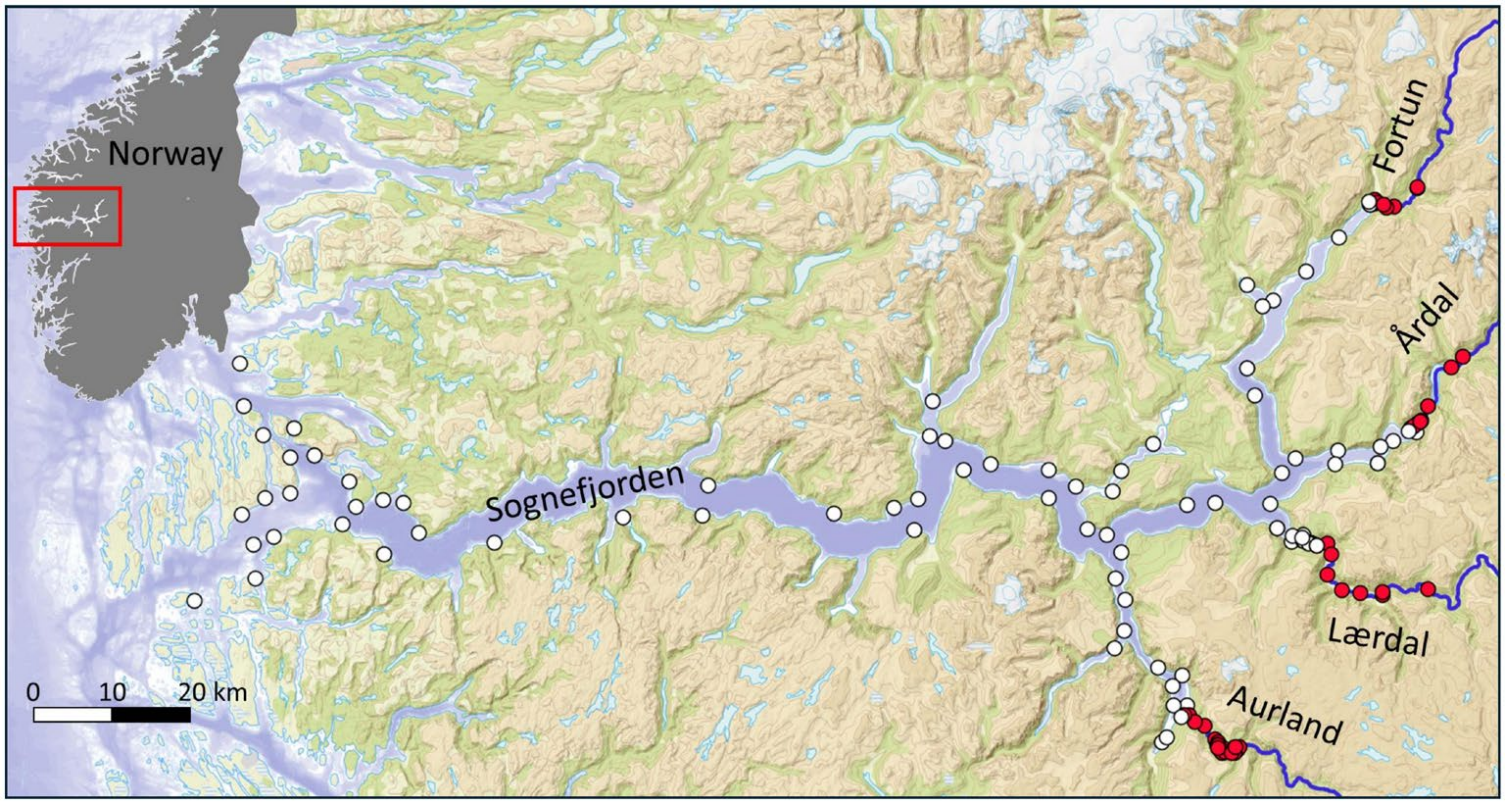
Locations of the four river arms included in the study. The insert illustrates the location of Sognefjorden on the west coast of Norway. White dots represent fjord receivers and red dots are freshwater receivers.

Sognefjorden is characterised by steep mountainsides, great depths and cold freshwater input from partly glaciated high‐altitude catchments. In its inner region, the fjord branches into several distinct fjord arms, each fed by freshwater systems supporting brown trout populations (Figure 1). This study included the four major rivers in this system. Three of these rivers (Aurland, Årdal and Fortun) contain lakes within the river stretches accessible to anadromous salmonids, and in these systems, brown trout is the dominant species. In Lærdal, the only study population without a lake, the Atlantic salmon stock has historically been the largest in the region, but this river also supports a population of anadromous brown trout.

In 1996, Lærdal was infected with the parasite *Gyrodactylus salaris* , leading to rotenone treatments in subsequent years. These treatments resulted in a near‐complete loss of juvenile salmonids and considerable numbers of larger brown trout overwintering in the river during this period. Despite this potential bottleneck, contemporary genetic differentiation of Atlantic salmon from this river has been shown to predate this rotenone treatment (Johnsen et al. 2014). Since the initial hydropower development in 1960, the intensity and range of anthropogenic alterations in Sognefjorden have increased substantially. The major impacts are summarised in Table 1.

**TABLE 1:** Summary of freshwater habitat, spawning population sizes (*N_c_*), harvesting pressure in freshwater, cultivation effort, hydropower impacts and aquaculture‐sourced salmon lice impact in the four study populations, all of which drain into Sognefjorden, Norway.

Population	Aurland	Lærdal	Årdal	Fortun
Catchment area (km^2^)	804	1184	981	508
Mean annual discharge (m^3^ s^−1^)	37.6	36.4	46.1	28.5
River length (km)	10	25	30	16
Lake area (km^2^)	1.9	0	7.5	0.62
Distance to fjord mouth from river mouth (km)	160	167	181	209
Spawning census counts (*N_c_*) (2003–2013) (mean *N* ± SD)	152 ± 72	680 ± 324	375 ± 186	336 ± 167
Annual riverine brown trout catch (2003–2013) (mean *N* ± SD)	211 ± 122	303 ± 200	207 ± 95	214 ± 122
Annual cultivation effort (smolt equivalents, mean *N* ± SD, years)	40,738 ± 21,445 (1979–2001)	9685 ± 6513 (1977–1996)	2988 ± 814 (1990–2012)	6576 ± 3625 (1990–2005)
Hydropower impact	Large	Medium	Medium	Large
Salmon lice impact	Low	Low	Low	Low

*Note:* Annual catch statistics derive from the national registry of river catches (http://www.lakseregisteret.no/). *N_c_* is estimated from visual surveys conducted during spawning seasons (see Table 3 for sources). Estimates of annual cultivation effort are based on information collected from local hatcheries by the authors or reported in Sægrov and Urdal (Sægrov and Urddal 2012, 2013). Calculation of smolt equivalents is based on Symons (1979) using a smolt age of 3.5 years and 2% survival from yolk sac fry to smolt. Hydropower and salmon lice impacts are based on assessments conducted by the Norwegian Scientific Advisory Committee for Atlantic Salmon (VRL 2022).

### 2.2 Acoustic Telemetry and Receiver Network

Acoustic telemetry is a biotelemetry system developed for aquatic organisms. Tags transmitting coded sound signals are implanted into the body cavity of the fish, and passive receivers distributed throughout the study system receive and store ID information. Detection occurs when tagged fish swim within the transmission range of a receiver. The date, time and unique identity of the fish are then transmitted and stored onto the detecting receiver. Hence, acoustic telemetry allows for in situ tracking of tagged individuals, from which statistical models targeting behavioural patterns can be built (Lennox et al. 2017).

A network of 138 acoustic receivers (VR2W; InnovaSea Systems Inc. Boston, U.S.) was deployed throughout the study period (September 2012 to December 2015) (Figure 1). The receiver network was designed to span the entire migration habitat from in‐river to open sea. A total of 44 receivers were located in freshwater, including 19 in lakes, while the remaining 99 were distributed throughout the fjord. Receiver locations were selected to maximise the detection probability of migrating tagged fish. The transmission range of the tags depends on the environment and tag power, ranging from a few meters within rivers and up to 1 km in fjord habitats (Ilestad et al. 2012). Data from the receiver network were downloaded approximately every 3 months to ensure continuous operation and prevent loss of data.

### 2.3 Fish Sampling

Veteran migrant brown trout, that is, fish that have previously undertaken at least one sea‐sojourn, were captured in freshwater using rod and line during September–October (2012–2014) and anaesthetised prior to surgery using tricaine methane sulphonate (MS‐222, 60 mg L^−1^, ca. 4 min immersion in aqueous solution). After reaching full anaesthesia, fish were placed ventral side up into a V‐shaped surgical tray with a continuous anaesthetic flow over the gills for the entire procedure (40 mg L^−1^ MS‐222). Tags were surgically inserted into the body cavity through a small incision posterior to the pelvic girdle, which was closed with three interrupted double surgical knots using a non‐absorbing 4/0 monofilament suture (www.resorba.com) and sealed with a tissue adhesive (monomeric n‐butyl‐2‐cyanoacrylate, Histoacryl). Scales were taken to estimate individual growth histories, and a small section of the pelvic fin was taken for DNA analyses and stored in 96% alcohol. When possible, fish were sexed by external appearance and features (*N* = 127). Typically, females exhibit smaller and rounder heads with blunt snouts, while males have larger, more angular heads with acute snouts. Males also develop a kype close to, during and for extended periods after spawning. Close to the spawning period, females will have a rounder body shape as their gonads occupy more space in the body cavity, and often an oviduct is visible in the vent. During this same period, males will display more pigmented and colourful bodies, and a larger adipose fin compared to similar‐sized females (Monet et al. 2006). The total length (TL) of the fish was also recorded, and total handling time was around 2 min per fish. The surgical procedure was conducted according to Urke, Kristensen, Arnekleiv, et al. (2013), Urke, Kristensen, Ulvund, et al. (2013), with approval granted by the Norwegian Animal Research Authority (ID 4638). Four different models of acoustic tags were deployed over the course of the study (LP‐9: 9 × 24 mm, 4 g; MP‐9‐SHORT: 9 × 24.4 mm, 3.6 g; AST‐9‐LONG 9 × 29.4 mm, 5.2 g and ADT‐13‐STAT: 12.7 × 33.3 mm, 7.1 g; Thelma Biotel AS, Trondheim, Norway). This study utilised a subset of the total tagged brown trout sample (Table 2). For further details on the tagging procedure and acoustic telemetry deployment see Hawley et al. (2024). All data analysis and modelling were conducted using R software version 4.3.3. (R Core Team 2024).

**TABLE 2:** Summary of the brown trout marked with acoustic tags during the study years 2012–2015 and genetically assigned to each sampled population of Sognefjorden.

Assigned population	Aurland	Lærdal	Årdal	Fortun
*N* brown trout sampled	55	37	27	23
TL (cm) (mean, range)	40.4 (24–72)	45.8 (25–73)	39.4 (25–62)	52.0 (28–81)
Sex ratio: male: female (*N*)	53:47 (49)	42:58 (36)	39:61 (23)	42:58 (19)
Smolt age (years, mean ± SD, (*N*))	3.09 ± 0.29 (22)	3.00 ± 0.47 (19)	2.58 ± 0.51 (19)	2.25 ± 0.45 (12)
Sea age at tagging (years, range)	1.7 (1–5)	1.5 (0–3)	1.5 (1–5)	2.3 (1–5)

Abbreviations: SD, standard deviation; TL, total length of fish at sampling.

### 2.4 Brown Trout Growth at Sea

Individual sea‐growth was estimated according to the maximum seaward extent reached during sea‐sojourn, with differential growth rates derived from empirical growth (scale readings) and acoustic telemetry data. A 6‐month period of sea growth was assumed, to estimate individual length at spawning (${\mathrm{TL}}_{\mathrm{Rep}}$) based on body length at sampling (TL), according to Hawley et al. (2024). The maximum seaward migration extent was determined as the watercourse distance (km) from the mouth of the sampling river and the furthest detecting receiver location. Each receiver was grouped into a fjord zone, dependant on spatial location within the fjord (inner, mid and outer fjord) (Hawley et al. 2024).

### 2.5 DNA Extraction and Genotyping

Genomic DNA was isolated from tissue samples (i.e., fin clips) according to the DNeasy Blood & Tissue kit (Qiagen) and adjusted to 50 ng/μL after determining concentration using the Qubit dsDNA BR Assay kit (Invitrogen). A total of 142 brown trout were genotyped using an Illumina iSelect SNP‐array containing 5509 brown trout SNP assays (Linløkken et al. 2017). Loci under selection were identified using BayeScan v2.1 (Foll and Gaggiotti 2008) and removed from the data set, along with monomorphic loci. Observed and expected heterozygosity were calculated using Arlequin v3.5.1.2 (Excoffier and Lischer 2010), and departure from Hardy–Weinberg equilibrium was tested at the 5% level after 1,000,000 Markov chain randomisations and 1,000,000 dememorization steps.

### 2.6 Genetic Population Assignment

Population admixture and clustering were determined by STRUCTURE v2.3.3 (Pritchard et al. 2000), utilising the admixture model with correlated allele frequencies (Falush et al. 2003), allowing individual genomes to represent a mixture of different populations. The number of clusters representing the data was selected from 20 independent runs, each consisting of 50,000 burn‐in iterations and 300,000 Markov chain Monte Carlo (MCMC) repeats, varying the cluster number between 1 and 10. The most likely number of homogenous clusters was evaluated based on Δ*K* (Evanno et al. 2005) and calculated using STRUCTURE HARVESTER (Earl and vonHoldt 2012). Additionally, the top level of hierarchical structure was further examined to assess potential subdivisions among the clusters. As with the top level, the most likely number of homogeneous clusters was determined using Δ*K*. For each K, the different runs were compiled, using the greedy function within CLUMPP v1.1.2 (Jakobsson and Rosenberg 2007) to maximise individual membership within each cluster.

To designate the most probable origin of all 142 brown trout specimens we implemented discriminant analysis of principal components (DAPC), using the R package *adegenet* (Jombart 2008). The number of clusters were defined a priori according to Δ*K* (from STRUCTURE). The optimal number of PCs to use was determined using the optim.a.score() command (e.g., Miller et al. 2020). Individual fish were then assigned to a population given the most probable posterior membership assignment, according to the DAPC output, irrespective of sampling location. Pairwise population measures of differentiation ($F_{\mathrm{ST}}$) (Weir and Cockerham 1984) were calculated using the R package *diveRsity* (Keenan et al. 2013) with significance tested by bootstrapping 1000 times. Linear regression of $\left(F_{\mathrm{ST}}/\left(1-F_{\mathrm{ST}}\right)\right)$ with waterway distance (calculated as shortest path between river mouths using QGIS) between population pairs (*N* = 6) was conducted to assess for correlation between genetic and geographic distances (Rousset 1997).

### 2.7 Evolutionary Dispersal and Gene Flow

Estimates of contemporary migration rates (m) between the four populations were calculated in BA3‐SNPs V 3.0.4 (Mussmann et al. 2019), an extension of the programme BayesAss3 (Wilson and Rannala 2003). This generates directional rates of gene flow using a Bayesian approach with MCMC sampling to estimate migration rates between each pair of populations, per generation (m). We ran the programme for 5 million iterations, with a burn‐in of 50,000 iterations and a sampling frequency of 100. Parameter space acceptance rates were between 20% and 60% (Wilson and Rannala 2003). To assess model convergence, we compared results from four replicate runs, each with a different random starting seed, where gene flow estimates were within ±0.05 across runs. We averaged the parameter estimates of the runs for subsequent analysis and estimated 95% credible intervals by calculating ±1.96 × standard error. The output can be presented in a *n* × *n* matrix; values on the diagonal are the proportion of resident genes derived from the source population during each generation (i.e., philopatric rate), values below the diagonal present rate of gene flow (*m*) from population *i* (where population *i* is the source) to population *j*, while values above the diagonal present *m* from population *j* to population *i* (where population *j* is the source).

Calculation of effective population size (*N*_*e*_) was conducted in NeEstimator v2.01 (Do et al. 2014) using the Linkage Disequilibrium method (Waples and Do 2008). To remove physically closely linked markers that may bias estimates of effective population size, all probes from the filtered SNP array were mapped to the current brown trout reference genome (fSalTru1.1, GCA_901001165.1, Hansen et al. 2021) using blast (BLAST+/2.14.1‐gompi‐2023a, Camacho et al. 2009). We then removed markers mapping to more than one location with greater than 95% identity, owing to the whole genome duplication event, as their physical position could not be unequivocally resolved. Once these were removed, assays within 100 kb of another SNP marker were removed. Following this filtering 2138 SNP markers remained with a mean distance between assays of 733,627 bp.

### 2.8 Identification of Potential Spawners‐Telemetry Data

The telemetry data had previously undergone a filtering process to identify erroneous or false detections (Hawley et al. 2024). Detections were deemed ‘false’ and removed if (a) they were at an improbable position in time and space relative to preceding detections, or (b) they occurred post expected battery expiration for a given tag. Potential spawning detections were identified as being in freshwater during the potential spawning period (week of year, WoY 35–52), for individuals with a ${\mathrm{TL}}_{\mathrm{Rep}}$ of > 34 cm (*N* = 84). Each detection was assigned a location (river: coded as 1–4), these locations were classified as either ‘philopatric’ (located in river of genetically assigned origin) or ‘dispersing’ (located in an alternate river than the river of genetically assigned origin). An individual could generate multiple dispersal locations within a given year, and an individual could be identified as both philopatric and dispersing during a given sampling year (*N* observations = 178, Table S1). We therefore stress that ‘dispersal’ (and philopatry) in this context is not necessarily permanent dispersal. Instead, this describes movement (or site‐fidelity) between river systems (i.e., ecological dispersal), with a potential to contribute towards the recipient spawning population (i.e., evolutionary dispersal) resulting in effective gene flow.

### 2.9 Modelling Potential Spawning Location

We ran categorical regression mixed‐effect models to generate the probability of potential spawning location. These included the fixed effects of population (genetic origin) and length (total length in cm at sampling, TL) of individual brown trout. We analysed the telemetry data using Bayesian mixed‐effects models fitted with the R package *brms* (Bürkner 2017), with all potential spawning locations (river: coded as 1–4) generated during all years of detection data set as the response variable (*N* obs = 178). Due to the repeated measures design of the data, with multiple locations generated per individual and year, individual fish ID was included as a random intercept. Models were run for 2000 iterations (1000 warmups) on four chains, using default priors. We performed posterior predictive checks to ensure adequate model fits, while trace plots confirmed that models converged with low among‐chain variability (Rhat = 1.00). We report posterior means with 95% credible intervals (CI) for all parameter estimates (Table S3). The fixed effect TL was scaled (mean = 0, SD = 1) to aid in model fitting and interpretation. Fits among models were compared by using leave‐one‐out cross validation information criterion (LOOIC) (Vehtari et al. 2017) (Table S2).

### 2.10 Modelling Dispersal Probability in Potential Spawners

We analysed dispersal probabilities (i.e., being detected as a potential spawner in a non‐natal river) using three different sets of generalised linear mixed‐effect models (*GLMER*) fitted using the *lme4* package in R (Bates et al. 2015). Model (a) included the fixed effects; genetic origin (population) and TL; model (b) genetic origin, TL and number of years since first sea migration (smolt), which increased annually over individual detection years (where age was determined from scale readings); and model (c) genetic origin, TL and maximum seaward migration extent of individuals that were detected within the fjord prior to the spawning period (during each detection year, km). Fish ID was included as a random intercept to account for repeated observations of individual fish (Zuur et al. 2009). The continuous effects TL and maximum migration extent were scaled to aid in model fitting and interpretation. Models were run independently for each data set, as the number of individuals for which each fixed effect was measured varied (Table S5). Genetic origin and TL were known for all 84 potential spawners (*N* obs = 178), maximum migration distance was generated for 55 spawners (*N* obs = 84) and age for 40 individuals (*N* obs = 79). For each set of models, the relative model support in the data was assessed using Akaike information criterion (AIC) (Anderson et al. 2001), adjusted for small sample size (AIC_c_) using the R package *AICcmodavg* (Mazerolle 2020) (Table S4). Each best performing model was then re‐run to include the fixed effect of sex, which was determined for a subset of 78 potential spawners (*N* obs = 172). We report all parameter estimates and standard error (SE) for each selected model (Table S5). McFadden's pseudo *R* ^*2*^ squared was calculated for each selected *GLMER* to compare the log likelihood of the full model (*L*) to the log likelihood of the model with just the intercept ($L_{0}$, the null model) according to: $R^{2}=1-\ln\left(L\right)/\ln\left(L_{0}\right)$.

### 2.11 Total Dispersal and Successful Spawning: Philopatry, Emigration and Immigration

Total numbers of successfully spawning individuals per generation were estimated from the predicted directional gene flow between each population pair (*m*) (generated in BayesAss3) (Figure 3), as a product of the source population effective population size (*N*_*e*_) (Table 4). Total philopatry and dispersal during the study period were estimated from the predicted probability of potential spawning location (Figure 3) as a product of the source population mean census count (*N*_*c*_) (Table 1), according to Equation i. These values were then scaled to match the number of breeding individuals (Equation ii) and further scaled to match the temporal period of gene flow estimates (*m*) (i.e., per generation, 5 years) according to Equation iii, where philopatry/dispersal estimates of fish from Aurland and Lærdal were generated over a three‐year period, Årdal and Fortun a two‐year period (Table S1).

- $N_{\text{Dispersal}}=\mathrm{Pr}\left(\text{Dispersal}\right)\times N_{c}$
- $\text{Total}N_{\text{Dispersal}}=N_{\text{Dispersal}}+(N_{\text{Dispersal}}\times \left(N_{c}/N_{e}))\right.$
- $\text{Total}N_{\text{Dispersal,}\;\mathrm{Generation}\;}=\left(\text{Total}N_{\text{Dispersal}}/3\right)\times 5$

Total out‐migration (emigration) and in‐migration (immigration) of potential spawners (ecological dispersal) and successful spawners (evolutionary dispersal, i.e., straying) were compared to determine the direction of net dispersal and gene flow between each population pair.

### 2.12 Population Growth Rate

An age‐structured Leslie matrix (Caswell 2000) was built to infer the intrinsic growth rate and stability of each population. This model is particularly suited to brown trout, as populations exhibit clear age structures due to limited growth during winter (Bærum et al. 2021), with census time selected so that reproduction occurs at the beginning of each annual period. The maximum age was set to 10 years, which corresponds to expected maximum age found in other systems in Norway (Jonsson 1985). The age‐structured matrix *A* includes two matrix elements; age class fecundities ($f_{a},a\;\epsilon \;\left\langle \alpha ,10\right\rangle$, where α is age at maturity), which form the top row of the matrix and age‐class survival ($S_{a}$), which form the diagonal of the matrix offset by one row:

$$\;A=\left[\begin{array}{ccccc} 0 & 0_{0} & f_{\alpha } & \cdots & f_{10}\\ S_{\text{init}}S_{0} & 0 & 0 & \ldots & 0\\ 0 & S_{1} & 0 & \ldots & 0\\ \vdots & \ddots & \ddots & \ddots & \vdots \\ 0 & 0 & 0 & S_{a-1} & 0 \end{array}\right]$$

Population‐specific estimates of $f_{a}$ and $S_{a}$ were generated from pre‐existing data, where methodologies were standardised across populations (Table 3). The demographic parameters supplying each matrix are presented in Table S6, with estimates generated from multiple years of sampling data (Table 3). The matrix was constructed using the *popbio* package in R (Stubben and Milligan 2007). From the Leslie matrices, we inferred the finite rate of population growth, lambda (*λ*). Population growth is stable when *λ* = 1, decreasing when *λ* < 1 and increasing when *λ* > 1. Given estimates of *λ* and net values of total evolutionary dispersal (successful straying) (Σm) we assigned each population pair as a ‘source’ or ‘sink’ according to: ${\mathrm{\Sigma m}}_{\mathrm{ij}}>{\mathrm{\Sigma m}}_{\mathrm{ji}}\&\lambda_{i}>\lambda_{j}$ then i = source and j = sink (and vice versa), alternatively if: ${\mathrm{\Sigma m}}_{\mathrm{ij}}>{\mathrm{\Sigma m}}_{\mathrm{ji}}\&\lambda_{i}<\lambda_{j}$ then i = weak source and j = weak sink, or the populations may be in equilibrium given sufficient deviation in the opposing directions (Kawecki 2004).

**TABLE 3:** Demographic information and literature sources used to construct Leslie matrices for each population (parameter estimates presented Table S6).

Parameter	Method	Pop.	Source, *N* years of data (period)
**Egg to age‐0 survival:** critical period ($S_{\text{init}}$)	The regression between log cohort‐specific number of eggs deposited (${\mathrm{Fec}}_{\mathrm{tot}}$) (estimated from spawning census, $N_{c}$) and density of 0+ in August same year (i.e., 2.5 months later)	AUR	Ugedal et al. (2023), 10 (2010–2020)
		LAR	Sættem (2016), 7 (2015–2021)
		ÅRD	Sægrov et al. (2013), 9 (2004–2013)
	$Z_{\text{init}}=\left[\ln\left({\mathrm{Fec}}_{\mathrm{tot}}\right)-\ln\left(N_{0+}\right)\right]/2.5$ $S_{\text{init}}=\exp\left(-Z_{\text{init}}\right)$	FOR	Sægrov et al. (2012), 6 (2006–2013)
**Juvenile survival** in freshwater, (dependant on smolt age) ($S_{0-2}$) or ($S_{0-3}$)	Catch‐curve analyses (Robson and Chapman, 1961) of cohort‐specific juvenile density data	AUR	Ugedal et al. (2023), 16 (2005–2020)
	For each population a linear mixed effects model was fitted:	LAR	Sættem (2020), 17 (1998–2019)
	log(density) ~ age + (1|Cohort)	ÅRD	Sægrov et al. (2015), 12 (2002–2013)
	The absolute value of the slope estimate for the age effect represents Z. $S_{0-3}=\exp\left(-Z_{0-3}\right)$	FOR	Hellen et al. (2016), 11 (2005–2015)
**Survival at sea** smolts and veteran migrants ($S_{3-10}$) or ($S_{4-10}$)	Simulations based on capture‐mark‐recapture multi‐state modelling (Conditional Arnason–Schwarz)	ALL	Hawley et al. (2024), 3 (2013–2015)
**Growth at sea** smolts: ($g_{\mathrm{SW}1})$ veteran migrants: ($g_{\mathrm{SW}2-7}$)	Directly measured from each population (fish scales)	ALL	Hawley et al. (2024), 3 (2013–2015)
**Fecundity** ($\bar{{\mathrm{Fec}}_{\mathrm{TL}}}$) ($f_{5-10}$)	${\mathrm{Fec}}_{\mathrm{TL}}=e^{-4.03+2.74*{\mathrm{TL}}_{\mathrm{Rep}}}$	ALL	L'Abée‐Lund and Hindar (1990)

## 3 Results

### 3.1 SNP Genotyping

A total of 5509 SNPs were initially used to genotype the samples. The average call rate for all samples was 0.998, with results reported from 4180 SNPs. From internal controls (retyping of 16 individuals, 11%), genotyping error rate was calculated to be negligible (0.003%). Further data cleaning removed 134 loci due to minor allele frequency (MAF), 241 loci due to monomorphism, 63 loci due to missing genotypes and 208 loci showed excess heterozygosity. Nine loci were found to be under selection and removed before continuing with the analyses. Mapping of the SNP loci to the brown trout reference genome assembly (fSalTru1.1, GCA_901001165.1, Hansen et al. 2021) identified 1387 loci being positioned closer than 100 kb to another SNP; these tightly physically linked loci were removed to ensure independence. Ultimately, 2138 loci were retained for the population genetic analyses.

### 3.2 Population Assignment

**FIGURE 2:**
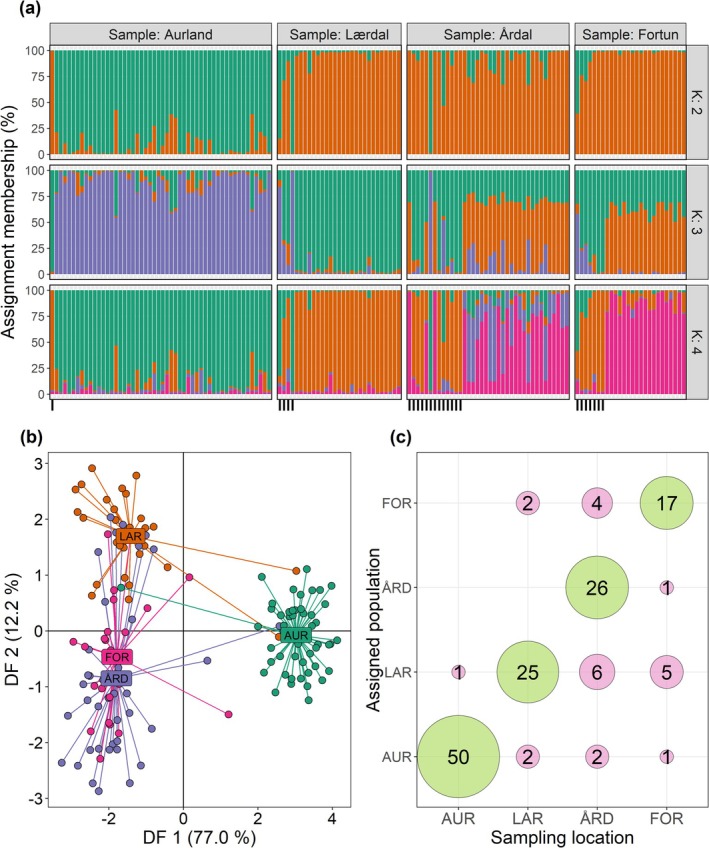
Population assignment of brown trout sampled from four populations of Sognefjorden (*N* = 142). (a) Results of clustering analyses generated in STRUCTURE, four clusters were identified (K), individuals are coloured according to their assignment membership (%) to each K. (b) Results of the discriminant principal component analysis (DAPC), showing discriminant function (DF) scores of each individual along the first two axes (77.0 and 12.2% of the variance explained, respectively). Individuals are coloured by sampling location. (c) Results of assignment tests, where green circles on the diagonal display the number of native individuals (where both sampling location and genetic assigned population match) and pink circles display the number sampled as dispersers (individuals sampled in an alternate river to which they are genomically assigned to). Circle size is proportional to the number of individuals in each category, the 24 brown trout identified as dispersers are indicated by a tick on the *x*‐axis of (a).

The delta‐K values generated from the STRUCTURE output revealed that the 142 brown trout were divided into two main groups: cluster 1, representing Aurland and cluster 2, encompassing the three remaining sampled populations. When these two clusters were analysed separately, cluster 2 was further subdivided into three subgroups denoting Lærdal, Årdal and Fortun (Figures S1 and 2a). These four groups were used as a priori clusters in the DAPC analysis, with the optimal number of PCs retained being 21 (Figure S2). The 1st discriminant function (DF) of the DAPC, distinguished the Aurland cluster and explained 77.0% of the variance, whilst the 2nd DF largely distinguished the remaining three populations, although considerable mixing among these populations was shown, with the variance explained reduced to 12.2% (Figure 2b). This structure was supported in the pairwise estimates of $F_{\mathrm{ST}}$, with Aurland most differentiated from the remaining three populations, and the degree of genetic isolation significantly correlated to waterway distance between population pairs (*RS* = 0.70, *F* = 4.48, *DF* = 4) (Table 4). The DAPC assignment of individuals into each population cluster revealed that 24 individuals were sampled as potential dispersers, with the greatest number sampled in Årdal (*N* = 12, Figure 2c). Estimates of *N*_*e*_ revealed small effective population sizes of brown trout. Only in Lærdal was *N*_*e*_ estimated to be greater than 500 individuals (*N*_*e*_ = 874), with *N*_*e*_ of Aurland brown trout the smallest, just 254 individuals (Table 4).

**TABLE 4:** Estimated effective population size (*N*_*e*_) (diagonal cells in italic font, 95% confidence intervals in parentheses), $F_{\mathrm{ST}}$ values (columns—blue shaded) and the shortest‐path waterway distance in kilometres (rows—grey shaded) between each pair of sampled populations of Sognefjorden.

		$F_{\mathrm{ST}}$ (95% CI)			
	**Population**	Aurland	Lærdal	Årdal	Fortun
**Distance (km)**	Aurland	*254* *(245–262)*	0.042 (0.034*–*0.051)	0.040 (0.033*–*0.046)	0.047 (0.040*–*0.056)
	Lærdal	59.6	*874* *(720–1111)*	0.022 (0.016*–*0.029)	0.024 (0.018*–*0.034)
	Årdal	75.2	35.0	*467* *(433–506)*	0.021 (0.015*–*0.029)
	Fortun	104.2	59.9	59.5	*316* *(290–347)*

### 3.3 Evolutionary Dispersal and Effective Gene Flow

**FIGURE 3:**
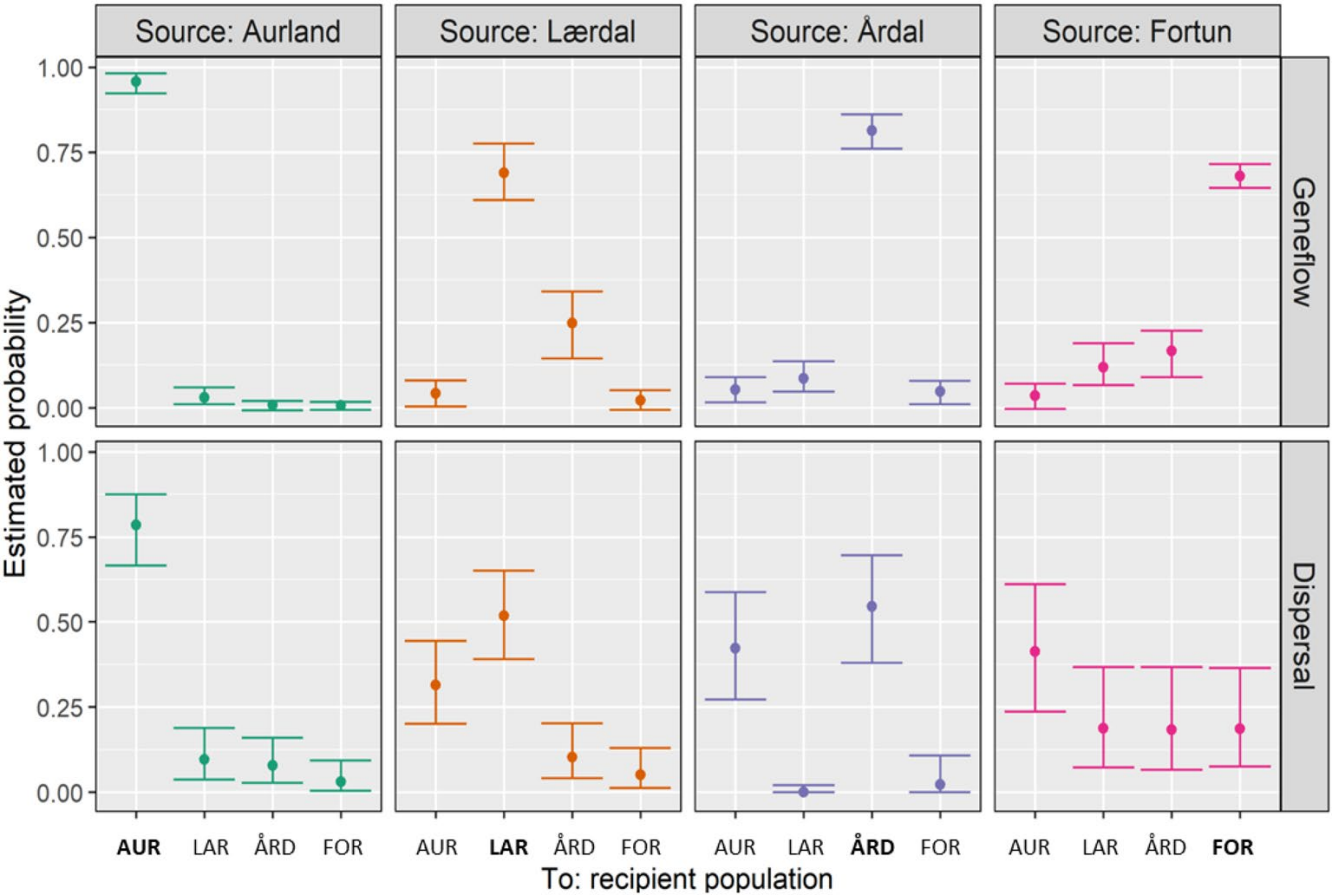
Estimated probability of gene flow (*m*) (top panel—evolutionary dispersal) and brown trout potential spawning location (bottom panel—ecological dispersal) between population pairs according to source population (top‐axis). Estimates were generated from the selected model in Table S3. Bold text on the *x*‐axis denotes where source and recipient population match (i.e., philopatry/homing), 95% credibility intervals are denoted by error bars.

Estimated rates of gene flow (m) were highest between Lærdal and Årdal ($m_{\mathrm{LAR}-\mathrm{ÅRD}}$ = 0.248), but asymmetric ($m_{\mathrm{ÅRD}-\mathrm{LAR}}$ = 0.086) (Figure 3). Unbalanced m was also estimated between Fortun and Årdal ($m_{\text{FOR}-\mathrm{ÅRD}}$ = 0.166, $m_{\mathrm{ÅRD}-\text{FOR}}$ = 0.047) and Fortun and Lærdal ($m_{\text{FOR}-\mathrm{LAR}}$ = 0.118, $m_{\mathrm{LAR}-\text{FOR}}$ = 0.022). Limited m was estimated from Aurland into the other three populations (range = 0.006–0.029), with estimates of m into Aurland higher for all populations (range = 0.035–0.052). In Aurland the probability of natal‐site fidelity was highest (0.957), with this reduced to 0.689, 0.814 and 0.680 for Lærdal, Årdal and Fortun, respectively (Figure 3).

### 3.4 Identification of Potential Spawners

A total of 84 tagged brown trout were identified as potential spawners, with these fish being detected in freshwater between the period WoY 35–52 and with a ${\mathrm{TL}}_{\mathrm{Rep}}$ of > 34 cm (Table 5). Most potential spawners were identified during 2013 (*N* = 69, Table S1), and originated from Aurland (*N* = 31, Table 5). A single individual spawner originating from Aurland was detected over a four‐year period, 63% of potential spawners were detected over 2 years, and 8% over three consecutive spawning seasons (Table S1). A total of 32 (38.1%) potential spawners were solely detected in their genetically assigned natal river, of which 19 (20.3%) were assigned to Aurland (Table 5). A total of 178 potential spawning locations (i.e., in freshwater during the potential spawning period) were identified, of which 101 (57%) were in the river of genetically assigned origin and 77 (43%) were in non‐natal rivers (Table S1). The origin and number of potential spawners identified and the location of each potential spawning detection during each year is stated in Table S1.

**TABLE 5:** Overview of the number of sampled brown trout identified as potential spawners in Sognefjorden, including those registered in natal (philopatric) and non‐natal rivers (dispersers).

Population	*N* dispersers (%)	*N* residents (%)	Total *N* potential spawners (% of total)
Aurland	12 (38.7)	19 (61.3)	31 (36.9)
Lærdal	19 (79.2)	5 (20.8)	24 (28.6)
Årdal	10 (58.8)	7 (41.2)	17 (20.2)
Fortun	11 (91.7)	1 (8.3)	12 (14.3)
**Total**	52 (61.9)	32 (38.1)	84

### 3.5 Dispersal Location Among Potential Spawners

The preferential model describing the potential spawning location included only the predictor natal river (genetically assigned population) (Figure 3 and Table S2). No effect of length or random intercept of Fish ID was included (Table S3). The model estimated a 79% probability for brown trout from Aurland to show site fidelity in potential spawning location, with low probabilities of dispersing to other rivers during the spawning period (0.10, 0.08 and 0.03, to Lærdal, Årdal and Fortun, respectively) (Figure 3). Conversely, all remaining populations had high probabilities of dispersing to Aurland: 0.32, 0.42 and 0.412, from Lærdal, Årdal and Fortun, respectively. The model generated low probabilities of Fortun as a potential spawning location, even among brown trout originating from Fortun (0.19), whereas dispersal probabilities of Fortun fish during the spawning season were high (0.41, 0.19 and 0.18, to Aurland, Lærdal and Årdal, respectively).

### 3.6 Traits Associated With Individual Dispersal Probability in Potential Spawners

**FIGURE 4:**
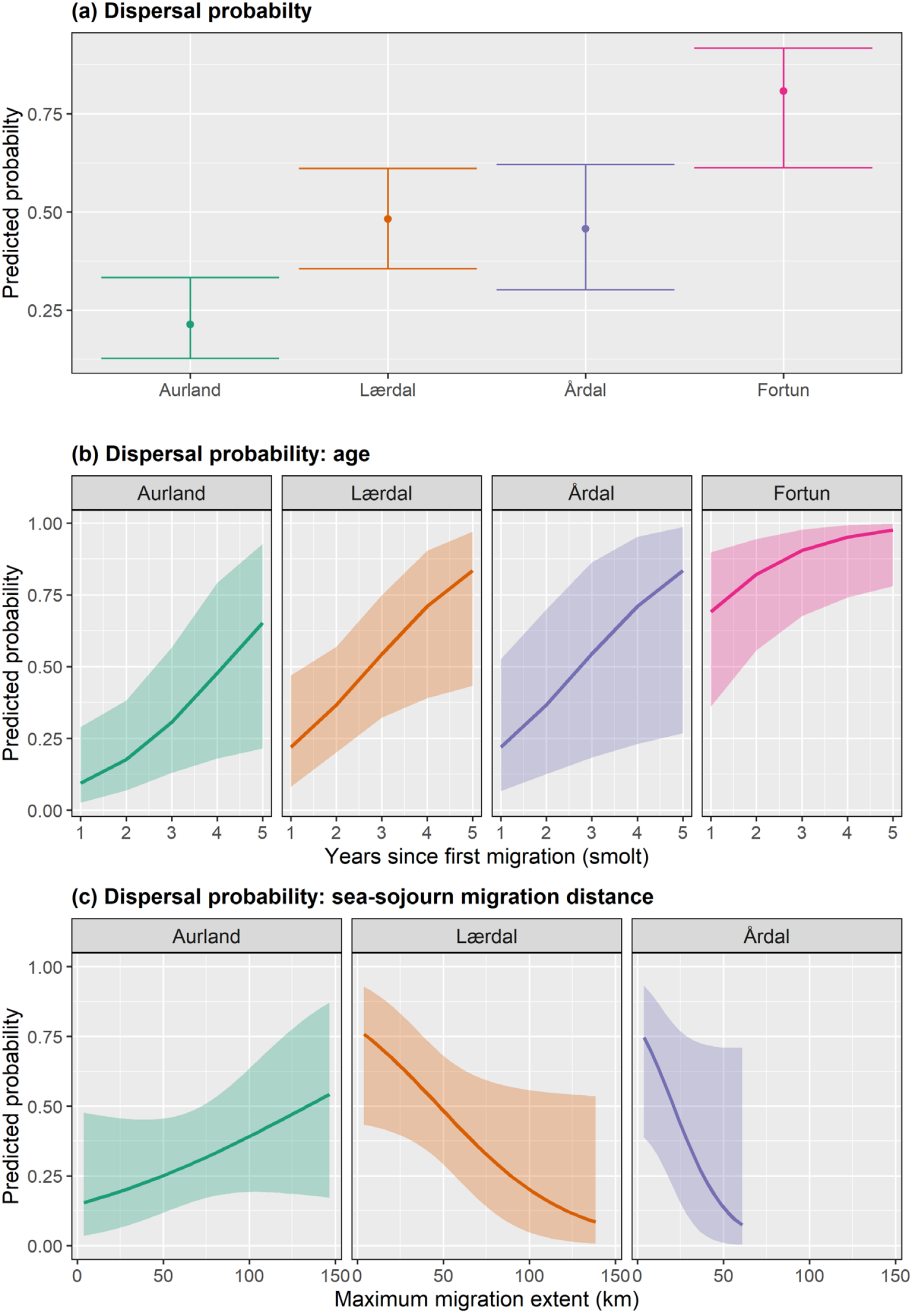
Dispersal probability of potentially spawning brown trout from Sognefjorden. (a) The effect of natal population on predicted ecological dispersal probability (*N* = 84), 95% confidence intervals are denoted by error bars. (b) Predicted dispersal probability according to the number of years since initial seaward migration (smolt) for each population (*N* = 40). (c) The effect of maximum seaward migration extent on predicted dispersal probability for each population (*N* = 55), where Fortun is not presented due to limited data leading to poor model fit for this population. All model parameters are reported in Table S5. Shaded regions denote 95% confidence intervals in all plots.

Despite repeated measurements of individual fish, the variance within individuals was estimated as zero for all three selected models describing the probability of dispersal among potential spawners. Therefore, the selected models were built as less‐complex *GLM*s (Table S4). The best‐performing model predicting the probability of dispersal among potential spawners only included the fixed effect natal river, with the predicted probability of dispersal highest for Fortun brown trout (Figure 4a, Tables S4 and S5a). In all populations, dispersal probability was predicted to increase with age, with the probability of dispersal again highest for brown trout from Fortun (Figure 4b, Tables S4 and S5b). An interaction effect of maximum seaward migration extent and natal population was selected to predict dispersal probability, with probability increasing with increasing migration distance for fish from Aurland. Conversely, dispersal probability decreased with increasing migration distance for fish from Lærdal and Årdal. Unfortunately, insufficient data resulted in poor predictive performance of the model for potential spawners from Fortun (Figure 4c, Tables S4 and S5c). We observed no effect of sex in any of the models predicting the probability of dispersal among potential spawners.

### 3.7 Total Ecological and Evolutionary Dispersal: Homing, Emigration and Immigration

**FIGURE 5:**
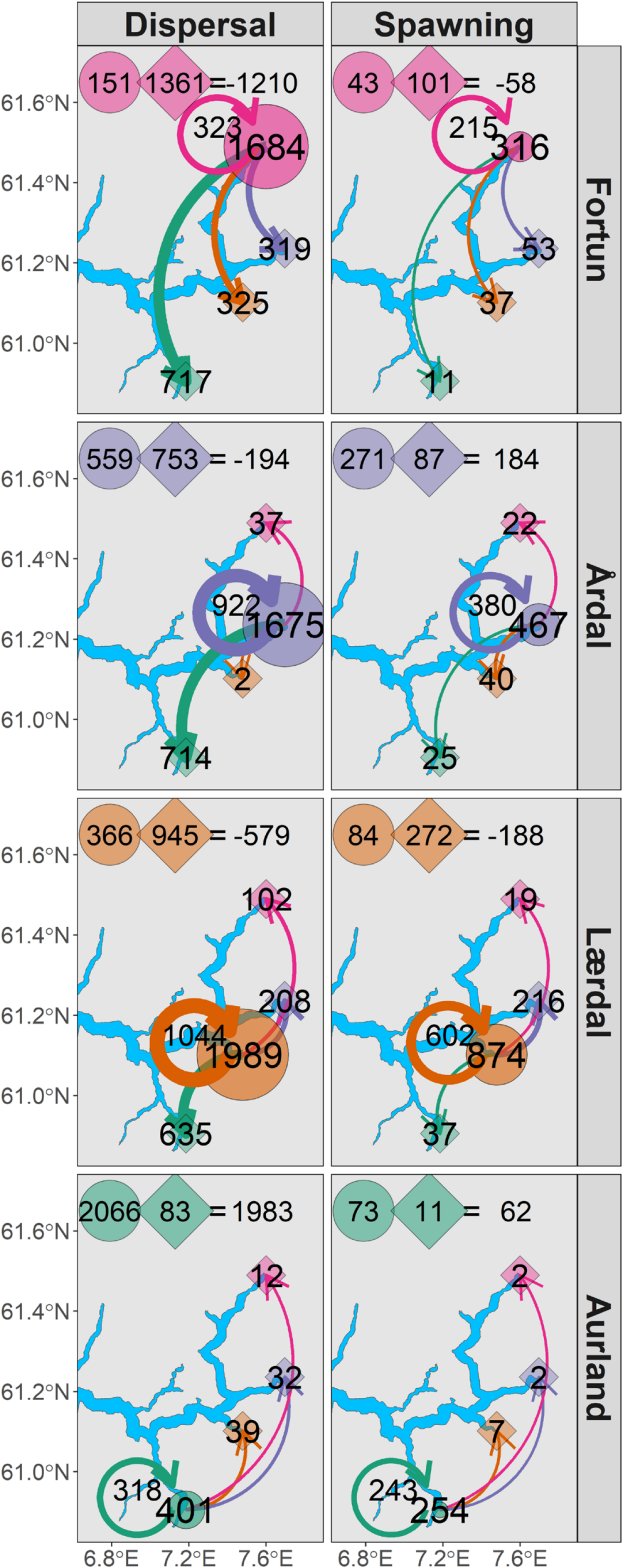
Estimated total ecological dispersal and effective spawning (evolutionary dispersal) numbers of brown trout sourced from each study population are represented by circles, with circle size proportional to the total number of individuals. Arrows from each source population are coloured by recipient populations (diamonds) and arrow thickness is proportional to the number of individuals estimated as dispersing/spawning from each source population (number stated within each diamond). The number of individuals returning to and spawning in their assigned natal population (philopatry/homing) is indicated within each circular arrow (colour matched to circle), and the total number of brown trout sourced from each population is stated within each circle. The sum of immigrants (circle) and emigrants (diamond) into and out of each population, along with the resulting net number of dispersers/spawners is stated at the top of each respective panel. A positive number indicates a gain of dispersers/spawners to a given location (i.e., total emigrating from < total immigrating to), while a negative number indicates a loss of individuals from a source population (i.e., total emigrating from > total immigrating to). The maps display the location of the four study rivers within Sognefjorden (blue shading). Total ecological dispersal during the 3‐year study period was estimated from the predicted probability of potential pawning location (Figure 3 and Table S3) as a product of the population census counts (*Nc*) (Table 1). These values were then scaled to match the temporal scale of gene flow estimates (i.e., per generation, 5 years) and the number of breeding individuals per generation (*Ne*). Estimates of total spawning between population pairs were generated according to predicted gene flow (*m*) × *N*_*e*_ (Table 4).

Total ecological dispersal was performed by 55% (3142 individuals) of the total population of potentially spawning individuals from all study populations per generation of brown trout (5749 individuals, Figure 5: dispersal panel). Total evolutionary dispersal resulting in gene flow was performed by 25% (471 individuals) of the total spawning population from all study populations, per generation of brown trout (1911 individuals, Figure 5: spawning panel). This equated to 15% of all dispersing individuals and 55% of natal homers successfully spawning per generation.

Lærdal and Årdal were identified as the largest populations in Sognefjorden, comprising 70% of the total brown trout spawning population per generation (Figure 5: spawning panel). Lærdal contributed 46% (874) of the total spawning individuals per generation, with 68% spawning in Lærdal (602 natal‐site homers, Figure 5: spawning panel). Among potentially spawning dispersers, 35% of total dispersers per generation were sourced from Lærdal (1989 individuals, Figure 5: dispersal panel).

Total brown trout dispersal resulted in a net gain of 1983 potential spawners into Aurland (immigrating into = 2066, emigrating from = 83), resulting in a gain of 62 spawning individuals per generation (immigrating into = 73, emigrating from = 11), with 3.5% and 13.3% of dispersing immigrants into and emigrants out of Aurland successfully spawning (Figure 5). In the remaining populations, estimated net ecological dispersal was negative (i.e., emigration > immigration); however, Årdal gained 184 spawning individuals. A net loss of 1210 emigrating potential spawners resulted in a net loss of 58 spawners per generation of Fortun brown trout. Total immigration resulted in effective gene flow from 8.9%, 4.7% and 2.7% of ecological dispersers per generation from Lærdal, Årdal and Fortun, respectively. Total emigration resulted in effective gene flow from 28.8%, 11.6% and 7.4% of ecological dispersers per generation from Lærdal, Årdal and Fortun, respectively.

### 3.8 Population Growth and Source–Sink Structure

The Leslie matrices estimated declining population growth (i.e., reproduction < mortality), for all populations except Fortun (*λ* = 0.777, 0.713, 0.862 and 1.205 for Aurland, Lærdal, Årdal and Fortun, respectively). Directional pairwise estimates of total evolutionary dispersal (straying) revealed the largest asymmetries were observed between Årdal and Lærdal ($\Sigma m_{\mathrm{ÅRD}-\mathrm{LAR}}$ = 40, $\Sigma m_{\mathrm{LAR}-\text{FOR}}$ = 216, Figure 5) and Fortun and Årdal ($\Sigma m_{\text{FOR}-\mathrm{ÅRD}}$ = 53, $\Sigma m_{\mathrm{ÅRD}-\text{FOR}}$ = 22, Figure 5), with Fortun projected as a source of dispersing brown trout to all populations (Table 6). Contrasting direction in the pairwise estimates of λ and *Σm* between Lærdal and Årdal/Aurland projected a weak or balanced source/sink structure between these populations (Table 6).

**TABLE 6:** Projected pairwise source–sink structure among brown trout populations of Sognefjorden, derived from estimates of total evolutionary dispersal (effective gene flow) and population growth rate (*λ*) between population pairs (columns: Recipient population, rows: Originating population).

Emigration from (source)	Population	Immigration to (recipient)			
		Aurland	Lærdal	Årdal	Fortun
	Aurland		Balanced	Sink	Sink
	Lærdal	Balanced		Weak source	Sink
	Årdal	Source	Weak sink		Sink
	Fortun	Source	Source	Source	

*Note:* Contrasting directions of *Σm* and *λ* reduce or negate the source–sink structure between population pairs, with source and sink populations being net exporters and importers of spawning brown trout individuals, respectively.

## 4 Discussion

### 4.1 Patterns of Ecological Dispersal Among Individuals and Populations

Ecological dispersal or movement among populations was prevalent among potentially spawning brown trout in Sognefjorden. We estimated that 55% of the total population of potential spawners from all study populations performed ecological dispersal (Figure 5). We observed that individual age and swimming distance during anadromy (seaward migration extent) affect the probability of undertaking dispersal movements within Sognefjorden (Figure 4) (H_1_).

In female iteroparous salmonids, fecundity, offspring growth and viability are improved with larger and older individuals (Nevoux et al. 2019). In Sognefjorden, brown trout undertaking long‐distance migrations are larger, with significantly greater fecundity than smaller, short‐distance migrants (Hawley et al. 2024). Thus, this association between migration extent and dispersal probability may have important demographic consequences. Limited numbers of long‐distance migrants would be required to generate substantial gene flow, particularly when recipient populations are small. Given that larger individuals undertake more extensive migrations, we would anticipate a positive association between dispersal probability and seaward migration extent. ‘Straying’ behaviour in salmonids is thought to occur more commonly among individuals undertaking greater migrations in both distance and duration (Quinn 1993; Keefer and Caudill 2014). However, this relationship was only observed among brown trout native to Aurland. A contrasting association, with decreasing dispersal probability as migration extent increased, was observed for anadromous individuals from Lærdal and Årdal (Figure 4). We suggest this to be a result of the hydroscape of Sognefjorden, with dispersal into Aurland, the outermost population and most frequently visited among dispersing individuals (Figure 5), occurring among those undertaking shorter‐distance migrations (i.e., remaining in the inner fjord in proximity of Aurland). In contrast, individuals reaching the mid‐outer fjord areas (> 100 km), bypass the fjord arm of Aurland (Figure 1), navigating seawards to the productive outer fjord region in the spring/summer and returning directly to natal spawning sites in the autumn. Such homing behaviour is generally considered the norm in anadromous salmonids (Quinn 1993; Keefer and Caudill 2014).

Veteran migrant trout display increased swimming activity while at sea. Their larger body size enhances swimming performance (Kennedy et al. 2022), enabling them to undertake exploratory movements with greater ease. We therefore expected dispersal movements to occur more frequently among larger individuals. However, our data revealed no effect of fish length on the probability of dispersal or on dispersal location. For a given individual detected over multiple years, dispersal (or philopatric) traits were not necessarily repeatable over consecutive years, with the likelihood for dispersal increasing with consecutive age for all populations (Figure 4). Thus, growth (i.e., consecutive length) would have been a superior predictor to include in the behavioural models, rather than the static length measurement taken at sampling. However, limitations in our data precluded our ability to robustly estimate this predictor. Few studies have explored traits associated with individual dispersal behaviour over consecutive years (but see e.g., Källo, Baktoft, Birnie‐Gauvin, et al. 2022) and our data imply that dispersal is a plastic life‐history trait that alters according to the age (and length) of the individual. We suggest this to be an interesting avenue for future research.

Collectively, dispersal movements occurred along a gradient from the innermost population (Fortun) towards the outermost population (Aurland). This resulted in a net loss (i.e., emigration > immigration) of potentially spawning brown trout from all populations except for Aurland (Figure 5). The population of Aurland was predominantly philopatric, with the proportion of non‐dispersing individuals approximately four times greater than the number of dispersers. In contrast, Fortun, the most migratory population, had a proportion of dispersers approximately three times greater than philopatric individuals (Figure 5). This disparity resulted in an asymmetric pattern of movement, with significant dispersal of potential spawners into Aurland and limited emigration of the native population.

We suggest that the hydroscape of Sognefjorden and the great distances required to swim to reach the productive outer fjord region (200 km) contribute to the observed direction of movement. Additionally, the closed structure of Sognefjorden presumably provides limited cause for migration in an inwards direction, although we did observe this direction of dispersal, albeit to a lesser degree. Consequently, anadromous brown trout from the innermost populations must swim past multiple fjord‐arms/river‐openings in both a seaward direction in the spring and an inland direction upon return for spawning (Figure 1), providing ample opportunity for dispersal events. Jonsson et al. (2018) suggest that anadromous brown trout may enter non‐natal rivers during the autumn, particularly during periods of high‐water flow, to feed, avoid osmotic stress, find shelter, or as a mechanism for removing marine ectoparasites, such as salmon lice. Hydropower regulations, which act in all rivers to varying degrees (Table 1), create stochastic aquatic environments with fluctuating water levels, flow and temperature. Overwintering in a heavily regulated river is therefore relatively risky for large anadromous brown trout individuals, with lake habitat or the fjord potentially providing more reliable overwintering habitat, as well as feeding habitat when an individual selects to forgo anadromy (Lennox et al. 2021). Thus, dispersal movements may be performed as a form of habitat selection or overwintering behaviour rather than true dispersal with implications for gene flow (i.e., spawning purposes). This form of dispersal behaviour has been observed among overwintering populations of the anadromous salmonid Arctic charr ( *Salvelinus alpinus* )(Moore et al. 2013, 2017). A study by Moore et al. (2017) showed that an asymmetric pattern of gene flow occurred in the opposite direction to migration movements of tagged fish. This implied that Arctic charr returned to their natal river to spawn but overwintered in rivers with the shortest migratory route to minimize migration costs in non‐breeding years. In Sognefjorden, the direction of dispersal movements from innermost to outermost populations implies that much of the dispersal performed by brown trout is a form of habitat selection in this extensive and directional hydroscape. This also provides an explanation for why we observed no differences in dispersal behaviour between the sexes of Sognefjorden brown trout. Among true dispersing individuals (i.e., with the intention to spawn), male‐biased dispersal is a widespread phenomenon (Bekkevold et al. 2004; Trochet et al. 2016; Li and Kokko 2019). However, we acknowledge that the number of individuals sexed in our data set was limited, as reliable visual sexing is often difficult to determine. Ideally, all fish would have been sexed based upon genetic data (e.g., Yano et al. 2013).

### 4.2 Evolutionary Dispersal: Spawning and Gene Flow

Dispersal generated contemporary gene flow among populations, with highest rates estimated between neighbouring populations and along a gradient, from the innermost to outermost populations (Figures 3 and 5). This pattern replicated the genetic variability across space, with the degree of genetic differentiation ($F_{\mathrm{ST}}$) correlated to distance between population pairs. Thus, the hydroscape and geographic distance affected the probability of evolutionary dispersal or straying among populations of Sognefjorden (H_2_).

A relatively high degree of genetic admixture was shown among the three innermost populations (Figure 2 and Table 4), with moderate to high levels of contemporary gene flow estimated among these populations (Figure 3). Conversely, limited genetic admixture was detected in Aurland brown trout. This population was the most genetically isolated, with low rates of contemporary gene flow (particularly emigration) between Aurland and the remaining three populations estimated. Values of total evolutionary dispersal showed the greatest straying success among neighbouring populations (Figure 5), with the process *isolation by distance* being a strong driver of genetic structuring in brown trout populations (e.g., Rodger et al. 2024). This implies that despite the high rates of dispersal movements observed in this system, philopatry and site fidelity still play an important role in the genetic structuring of brown trout populations in Sognefjorden. We estimated that successful dispersal (straying) was performed by 25% of the total spawning population; thus, 75% of total spawning success occurred in natal rivers.

We estimated that 15% of all dispersing individuals and 55% of natal homers successfully spawned per generation. Few studies have directly compared the rate of ecological dispersal versus evolutionary dispersal in brown trout, limiting our opportunity for comparison. However, Masson et al. (2018) measured almost no gene flow resulting from the immigration of anadromous brown trout within a series of dendritic river systems. In contrast, Källo et al. (2023) were unable to distinguish individual populations within a small fjord system due to high rates of dispersal (35%–55%) and accompanying gene flow. However, the authors observed that the populations within the study fjord were genetically discernible from neighbouring fjord anadromous brown trout populations. Among populations, we observed considerable differences in the proportion of overall immigration and emigration dispersal movements that resulted in successful spawning. For example, very high numbers of individuals dispersing into Aurland (2066) resulted in just 3.5% (73 individuals) of immigrants successfully contributing to the next generation of brown trout. In contrast, just under half of the total migrants into Årdal (48.5%, *N* = 271) successfully contributed to gene flow into this population. This resulted in a net gain of 184 spawning brown trout per generation. Unfortunately, we are unable to state whether the anomaly between rates of dispersal movement and successful dispersal (straying) is due to habitat selection (i.e., over‐wintering or position of the populations within the fjord) or unsuccessful spawning events among true dispersing individuals (or a combination of the two). One known limitation to gene flow between populations is selection processes acting against strayers and their offspring (Mobley et al. 2019). Natal philopatric (homing) individuals may have a reproductive fitness advantage resulting from pre‐ and postzygotic spawning processes, including habitat selection, mate choice, assortative mating and reduced fitness of hybrid offspring. Consequently, lower reproductive fitness of dispersers may act as a reproductively isolating mechanism, which in turn develops local adaptations (Peterson et al. 2014; Mobley et al. 2019).

Given directional net gene flow from innermost to outermost populations, we would anticipate greater genetic admixture in the outermost population (Aurland) relative to the inner populations. Instead, our data present a paradox, with greater genetic admixture among the three innermost populations and relative genetic isolation with limited admixture among brown trout native to Aurland. This potentially implies reduced fitness of hybrid offspring in Aurland, with these individuals less likely to contribute to the next generation of spawners due to poor survival rates, as seen in similar lake systems (Wollebæk et al. 2012). Postzygotic selection against hybrids, in combination with genetic drift, which in this small population (*N*_*e*_ = 254) may operate to a significant degree, could account for the relative genetic isolation of this population. Lakes have been shown to limit gene flow among catchments, with the success of spawning migrations of adult fish and downstream migration of smolts through lakes thought to have an adaptive basis (Aarestrup and Koed 2003; Dillane et al. 2008). Consequently, lakes can act as landscape features that shape genetic population structure by restricting within‐river migration (Dillane et al. 2008). The large lake in Aurland, Vassbygdvatnet, may therefore also contribute to the relative genetic isolation of this population.

### 4.3 Source–Sink Structure and Implications for Population Management

Directional (i.e., unbalanced) patterns of pairwise evolutionary dispersal (successful straying) combined with discrete estimates of intrinsic population growth have generated a source–sink structure among brown trout populations of Sognefjorden (Table 6) (H_3_). The Leslie matrices revealed that only Fortun exhibits intrinsic population growth (*λ* = 1.2). In the remaining populations, the rate of estimated mortality was greater than that of reproduction (*λ* < 1). As a result, Sognefjorden is comprised of multiple sink populations being supplemented by dispersing brown trout individuals, primarily from a single source population, Fortun (Table 6). Contrasting directions in the pairwise estimates of λ and the total net number of successful strays generated a more complex source–sink structure among the remaining three populations. For example, Årdal exported spawning individuals to Aurland, acting as a source, but was conversely projected as a weak sink for spawning brown trout from Lærdal. Thus, the extensive hydroscape and the distances between populations likely contribute to the formation of Sognefjorden's source–sink structure.

In a metapopulation, the availability of recruits depends on the source population's compensatory reserve (Pulliam 1988). Consequently, metapopulation persistence is dependent upon an excess compensatory reserve in its source populations, with a sufficient reserve required to compensate for stochastic effects on its own mortality (Kaeding 2023). In Fortun, high rates of estimated survival during the anadromous life‐stages (i.e., smolt and veteran migrant) account for the excess of available recruits in this population (Table S6). Although the reasons for this superior survival are unclear, it has been estimated that the proportion of autumn migrants, that is, return to the fjord to overwinter post‐spawning, and the proportion of skipped spawners are highest in this population (Hawley et al. 2024). This behaviour may generate superior feeding opportunities when compared to remaining in freshwater in an energetically depleted condition post‐spawning (Lennox et al. 2021). This strategic behaviour may also act as a form of energy conservation in iteroparous salmonids (Birnie‐Gauvin et al. 2023), a strategy that may have been selectively favoured for this population, due to the highest migration costs from its innermost location within the fjord. Successful dispersal from this population into neighbouring connected population demes may therefore play an important role in providing genetic variance pertinent to fjord use and survival given contemporary conditions within the fjord. We therefore maintain that monitoring of Fortun's brown trout population should be prioritised within Sognefjorden, with management directed at sustaining genetic variation among the potential recruits from this population.

In Sognefjorden, multifaceted disturbances have contributed to a significant reduction of brown trout in recent times, with previous levels of harvesting in freshwater beyond sustainable limits and the extent of harvest at sea largely unknown (Table 1). A shortcoming of current management strategies for salmonids is that neither conservation nor optimal harvest criteria are well developed for groups of populations interconnected by gene flow (Hindar et al. 2004; Schtickzelle and Quinn 2007). Under a metapopulation paradigm, management criteria should be based not only on the population size and demography of the local population but also on the connected demes and the level of dispersal and gene flow between them (Hindar et al. 2004; Schtickzelle and Quinn 2007). However, the conservation benefit of a metapopulation structure is uncertain for populations of low productivity, with remediation that instead increases population‐specific demography thought to be more effective (Hastings and Botsford 2006; Bowlby and Gibson 2020). Nevertheless, dispersal may result in populations that are better able to respond to environmental change, provided it generates sufficient gene flow to maintain or increase genetic variance and therefore adaptive potential, but without eliminating local adaptations (Peterson et al. 2014).

In conclusion, our study reveals how an interdisciplinary approach identified traits associated with individual dispersal movements and gene flow, which resulted in biologically relevant population structuring at the metapopulation scale. Unfortunately, our study only provides a contemporary outlook, lacking long‐term population dynamics data. The use of archived samples, such as historical fish scales (Østergaard et al. 2003; Thaulow et al. 2013, 2014) and otoliths (Ruzzante et al. 2001; Källo et al. 2023) has made it feasible to study the genetic composition of populations over several decades. We suggest employing such methods (given relevant samples are accessible) to gain insights into the historical genetic structuring of Sognefjorden brown trout. This would yield valuable information on metapopulation *dynamics* over an evolutionary timeframe in this heavily altered but unique system. Such knowledge would contribute towards improved models of population management for connected demes of anadromous fish.

## Conflicts of Interest

The authors declare no conflicts of interest.

## Supporting information


Data S1.

## Data Availability

The data that support the findings of this study (Hawley et al. 2025) are available from DataverseNO (https://doi.org/10.18710/IGV7XU).
