# Supplementary material for: Dispersal and Gene Flow Among Potential Spawners: Source–Sink Structure Among Populations of Anadromous Brown Trout Exposed to Multifaceted Anthropogenic Impacts
Source: Evol Appl. 2025 Jul 8;18(7):e70130. doi: 10.1111/eva.70130 (PMC12238681; doi:10.1111/eva.70130)
Supplement: Supplementary file 1 — Data S1. [file EVA-18-e70130-s001.pdf]

Dispersal and gene flow among potential spawners: source-sink structure among populations of anadromous brown trout exposed to multi-faceted anthropogenic impacts.

K. L. Hawley[1,2*], J. Thaulow[1], H. A. Urke[3], T. Kristensen[4], N. J. Barson[5] and T. O. Haugen[2]

[1] Norwegian Institute for Water Research, Økernveien 94, 0579 Oslo, Norway

[2] Faculty of Environmental Sciences and Natural Resource Management, The Norwegian University of Life Sciences, Høgskoleveien 12, 1433 Ås, Norway

[3]AquaLife R&D, Havnegata 9, 7010 Trondheim, Norway

[4]Faculty of Biosciences and Aquaculture, Nord University, Universitetsalléen, 8049 Bodø, Norway

[5]Faculty of Biosciences, Norwegian University of Life Sciences, Oluf Thesens vei 6, 1433 Ås, Norway

Supplementary Results

Table S1: The number of potentially spawning brown trout individuals genomically assigned to each source population of Sognefjorden, for each sampling year. The number and location of potential spawning detections are also stated.

Detection year	Native population	N fish	Dispersal location (N detections)			
			Aurland	Lærdal	Årdal	Fortun
**2012**	Aurland	12	**12**	0	0	0
	Lærdal	1	1	**0**	0	0
	Total	*13*	*13*	*0*	*0*	*0*
**2013**	Aurland	21	**20**	3	2	0
	Lærdal	22	9	**16**	5	2
	Årdal	16	10	0	**14**	1
	Fortun	10	7	3	4	**5**
	Total	*69*	*46*	*22*	*25*	*8*
**2014**	Aurland	17	**13**	1	3	2
	Lærdal	17	8	**11**	1	1
	Årdal	10	5	0	**5**	0
	Fortun	8	4	2	1	**0**
	Total	*52*	*30*	*14*	*10*	*3*
**2015**	Aurland	5	**3**	2	0	0
	Lærdal	2	0	**2**	0	0
	Total	*7*	*3*	*4*	*0*	*0*

Table S2: Expected log predictive density scores (elpd) for each Bayesian categorical regression model generated to investigate the potential spawning location of Sognefjorden brown trout.

Response	Model list	Δ elpd	Δ SE
Potential spawning location (River:1 – 4) (Categorical regression model)	~ Population	0	0
	~ Population + TL	-0.2	2.8
	~ Population + (1|FishID)	-2.4	2.2
	~ Population + TL + (1|FishID)	-3.9	3.5
	~ TL	-26.3	8.9
	~ TL + (1|FishID)	-26.8	8.8

Note: Models were ranked according to the differences in expected log predictive density (Δ elpd) from the model of best fit (i.e. the model with the lowest leave-one-out-cross-validation information criterion (LOOIC)). (1|FishID) denotes that fish ID was included as a random intercept, to account for repeated observations of individual fish. Total fish length at sampling is expressed by TL (standardised). SE = standard error.

Table S3: Potential spawning location of brown trout model statistics.

Response	Variable	Estimate	Est. Error	Lower 95 % CI	Upper 95 % CI	Rhat	ESS
Potential spawning location (River:1 – 4)	To Lærdal (Intercept)	-17.10	16.70	-62.08	-2.92	1.01	510
	To Årdal (Intercept)	0.26	0.35	-0.43	0.93	1.00	2381
	To Fortun (Intercept)	-3.18	1.32	-6.42	-1.19	1.00	1082
	To Lærdal: From Aurland	14.98	16.72	0.72	60.05	1.01	506
	To Lærdal: From Fortun	16.30	16.72	1.94	61.31	1.01	508
	To Lærdal: From Lærdal	17.61	16.70	3.34	62.53	1.01	510
	To Årdal: From Aurland	-2.59	0.62	-3.84	-1.46	1.00	2155
	To Årdal: From Fortun	-1.09	0.69	-2.49	0.19	1.00	2092
	To Årdal: From Lærdal	-1.38	0.61	-2.63	-0.20	1.00	2430
	To Fortun: From Aurland	-0.18	1.54	-2.89	3.32	1.00	1150
	To Fortun: From Fortun	2.37	1.43	-0.02	5.81	1.00	1059
	To Fortun: From Lærdal	1.31	1.49	-1.19	4.80	1.00	1122

Note: Bayesian categorical regression model compares the probability of potential spawning location (1 – 4) for each study population of Sognefjorden (1 – 4), N = 84, observations = 178. CI = Bayesian credible interval, Rhat = Gelman-Rubin statistic, ESS = effective population size.

Table S4: AIC scores of the models generated to investigate the dispersal and homing behaviour of potential brown trout spawners in Sognefjorden.

Model	Model list	K	AICc	Δ AICc
**(a) Dispersal- all fish** *GLMER*	~ Population + (1|FishID)	5	224.8	0.0
	~ TL * Population + (1|FishID)	9	225.9	1.1
	~ TL + Population + (1|FishID)	6	226.9	2.1
	~ TL + (1|FishID)	3	248.1	23.2
**(b) Dispersal- age** *GLMER*	~ Population + Sea age + (1|FishID)	6	97.3	0.0
	~ Population * Sea age + (1|FishID)	9	99.0	1.7
	~ TL + Population + Sea age + (1|FishID)	5	99.2	2.0
	~ Population * Sea age + (1|FishID)	6	99.8	2.5
	~ Population + (1|FishID)	5	100.6	3.4
	~ TL * Population + (1|FishID)	9	101.4	4.2
	~ Sea age + (1|FishID)	3	107.1	9.8
	~ TL + (1|FishID)	4	107.5	10.2
	~ TL + Sea age + (1|FishID)	4	107.8	10.6
	~ TL * Population * Sea age + (1|FishID)	17	119.1	21.8
**(c) Dispersal- migration distance** *GLMER*	~ Population * Mig. Distance + (1|FishID)	9	111.3	0.0
	~ Population + (1|FishID)	5	116.3	5.1
	~ Population + Mig. Distance + (1|FishID)	6	116.9	5.6
	~ TL + Population + Mig. Distance + (1|FishID)	7	119.2	8.0
	~ TL * Population * Mig. Distance + (1|FishID)	17	120.8	9.5
	~ Mig. Distance + (1|FishID)	3	121.0	9.8

Note: In all instances the principle of parsimony was adhered to and the model with the lowest AIC score was selected. ΔAICc denotes the difference between a candidate model's AICc value compared to the one with the lowest AICc, K states the number of model parameters. (1|FishID) denotes that fish ID was included as a random intercept, to account for repeated observations of individual fish (generalised liner mixed models- *GLMER*). Total fish length at sampling is expressed by TL, continuous variables (TL and migration distance) were standardised.

Table S5: Summary statistics and parameter estimates from the models used to fit the dispersal and homing probability data of potential brown trout spawners in Sognefjorden.

Model	N FishID	N obs	R^2	Term	Estimate	SE
**(a)**	84	178	0.119	Intercept	-2.171	0.339
				Aurland	-1.134	0.461
				Fortun	1.606	0.602
				Lærdal	0.100	0.432
**(b)**	40	79	0.217	Intercept	-1.992	0.827
				Aurland	-0.989	0.918
				Fortun	2.074	0.956
				Lærdal	-0.001	0.846
				Years since smolt	0.7224	0.322
**(c)**	55	84	0.217	Intercept	-2.275	1.617
				Aurland	1.270	1.675
				Fortun	2.558	2.263
				Lærdal	2.028	1.675
				Mig. distance	-2.583	1.457
				Mig. distance *Aurland	3.116	1.518
				Mig. distance * Fortun	22.405	31.197
				Mig. distance * Lærdal	1.512	1.556

Note: Models were selected according to AIC (Table S4). *SE* denotes the standard error for the estimates. R^2 gives the McFadden's' R-squared value of the model. Total fish length (at sampling) is denoted by *TL*. Continuous variables (*TL* and migration distance) were standardised. Backwards selection was undertaken to remove non-significant interaction effects in selected models, to extend the principle of model parsimony.

Table S6: The demographic parameters included in each population-specific Leslie matrix. For age 0 survival was split into critical period survival and 0 + survival. The critical period was defined as time between swim-up (early June) and late August (i.e., 2.5 months). By assuming no egg-to-swim-up mortality, critical period instantaneous mortality (Z_{init}) could be estimated from $[\ln(Fec_{tot}) - \ln(N_{0+})]/2.5$ then corresponding survival as $S_{init} = \exp(-Z_{init})$.

Population	Life stage	Age	Growth	Length	Fecundity	Survival
**Aurland**	Critical period	0			0	$3.75e^{-6}$
	0+	0			0	0.55
	Parr	1			0	0.55
	Parr	2			0	0.55
	Parr	3			0	0.55
	Smolt	4	0.654	20.1	0	0.45
	Finnock	5	0.412	27.9	0	0.77
	Kelt	6	0.412	34.3	816	0.77
	Kelt	7	0.412	42.1	1435	0.77
	Kelt	8	0.412	51.7	2525	0.77
	Kelt	9	0.412	63.6	4441	0.77
	Kelt	10	0.412	78.2	7812	0.77
**Lærdal**	Critical period	0			0	$3.76e^{-5}$
	0+	0			0	0.27
	Parr	1			0	0.27
	Parr	2			0	0.27
	Parr	3			0	0.27
	Smolt	4	0.697	19.5	0	0.52
	Finnock	5	0.346	27.6	0	0.68
	Kelt	6	0.346	32.9	728	0.68
	Kelt	7	0.346	39.1	1170	0.68
	Kelt	8	0.346	46.5	1879	0.68
	Kelt	9	0.346	55.2	3020	0.68
	Kelt	10	0.346	65.7	4854	0.68
**Årdal**	Critical period	0			0	$3.83e^{-7}$
	0+	0			0	0.32
	Parr	1			0	0.32
	Parr	2			0	0.32
	Smolt	3	0.654	15.6	0	0.64
	Finnock	4	0.412	21.6	0	0.86
	Kelt	5	0.412	26.6	0	0.86
	Kelt	6	0.412	32.7	717	0.86
	Kelt	7	0.412	40.2	1261	0.86
	Kelt	8	0.412	49.3	2217	0.86
	Kelt	9	0.412	60.6	3899	0.86
	Kelt	10	0.412	74.5	6858	0.86
**Fortun**	Critical period	0			0	$2.74e^{-3}$
	0+	0			0	0.29
	Parr	1			0	0.29
	Parr	2			0	0.29
	Smolt	3	0.610	18.2	0	0.75
	Finnock	4	0.470	24.7	0	0.91
	Kelt	5	0.470	31.2	0	0.91
	Kelt	6	0.470	39.5	1206	0.91
	Kelt	7	0.470	50.0	2297	0.91
	Kelt	8	0.470	63.2	4373	0.91
	Kelt	9	0.470	80.0	8326	0.91
	Kelt	10	0.470	101.2	15853	0.91

Figure S1: Delta K values estimated according Evano et al. (2005) and generated in STRUCTURE, where figure **(a)** includes individual brown trout (N =142) from all four sampled populations and **(b)** excludes individuals sampled from the population Aurland.

(a) Delta K: All four populations included

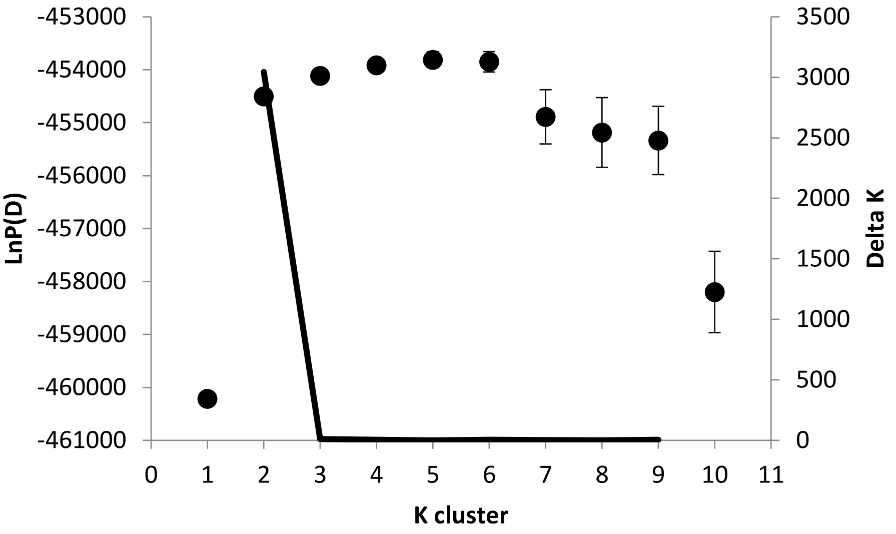

(b) Delta K: Three populations (Aurland excluded)

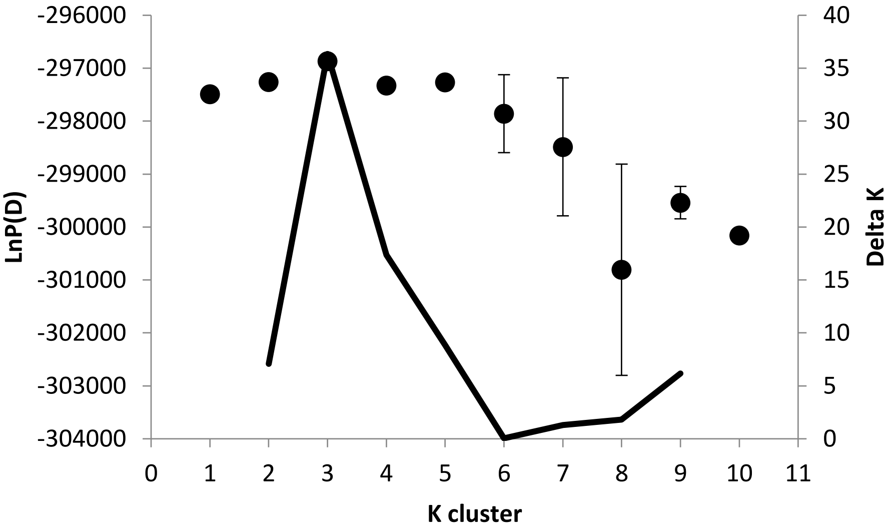

Figure S2: The optimal number of principal components (PCs) to retain in the discriminant principal component analysis (DAPC) used to assign individual brown trout (N=142) into four population clusters (as defined a priori using STRUCTURE). The optimal number was estimated by the optim.a.score() command in the R package *adegenet* (Jombart 2008).

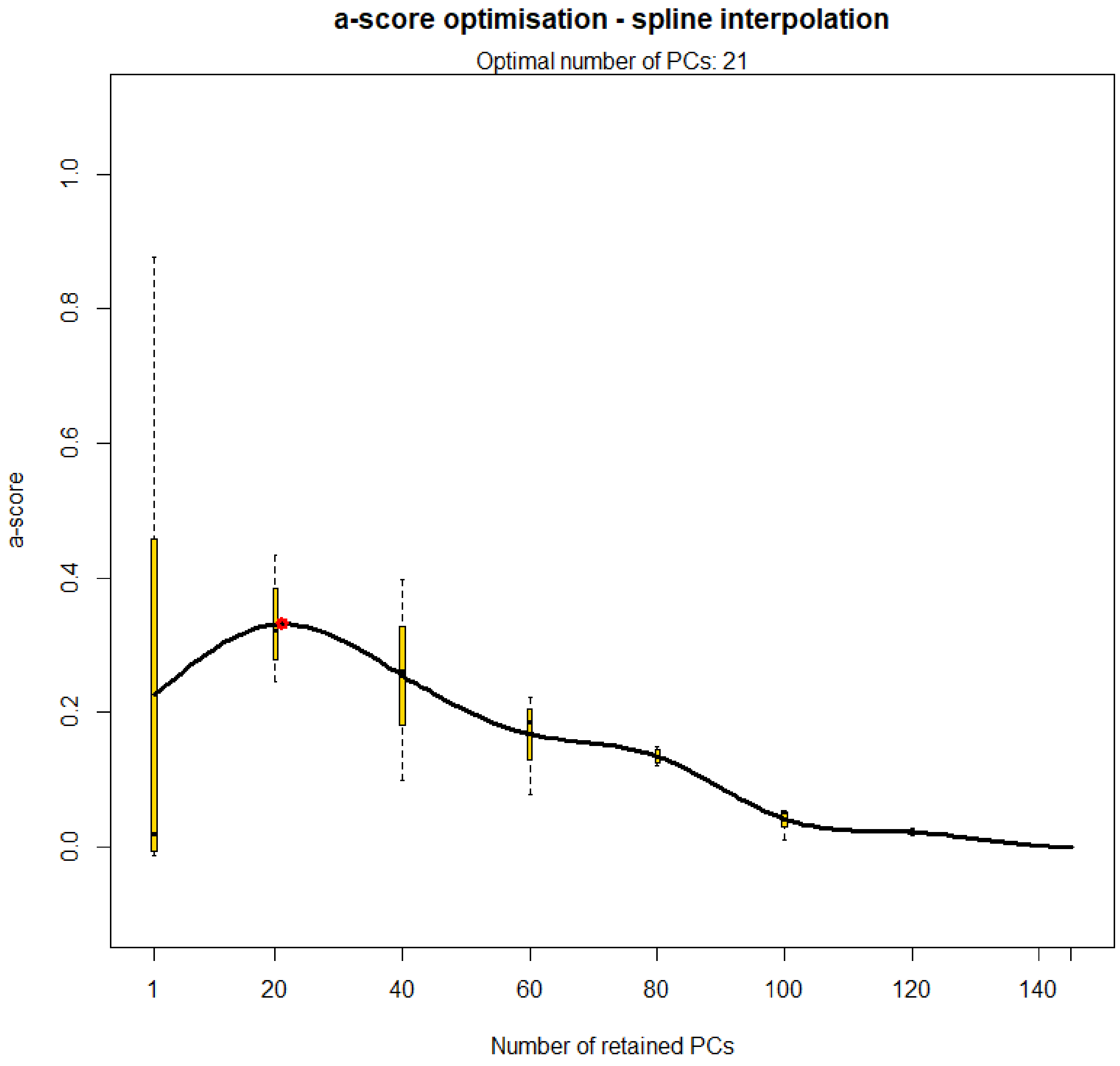

References

Evanno G., S. Regnaut and J. Goudet. 2005. Detecting the number of clusters of individuals using the software STRUCTURE: a simulation study. Mol Ecol 14:2611–2620

Jombart, T. 2008. adegenet: a R package for the multivariate analysis of genetic markers. Bioinformatics 24:1403–1405.
